# The agricultural antibiotic carbadox induces phage-mediated gene transfer in *Salmonella*

**DOI:** 10.3389/fmicb.2014.00052

**Published:** 2014-02-11

**Authors:** Bradley L. Bearson, Heather K. Allen, Brian W. Brunelle, In Soo Lee, Sherwood R. Casjens, Thaddeus B. Stanton

**Affiliations:** ^1^Agroecosystems Management Research Unit, National Laboratory for Agriculture and the Environment, ARS, USDAAmes, IA, USA; ^2^Food Safety and Enteric Pathogens Research Unit, National Animal Disease Center, ARS, USDAAmes, IA, USA; ^3^Department of Biological Sciences and Biotechnology, Hannam UniversityDaejeon, South Korea; ^4^Division of Microbiology and Immunology, Department of Pathology, University of UtahSalt Lake City, UT, USA

**Keywords:** *Salmonella*, bacteriophage, antibiotic, carbadox, prophage, gene transfer, transduction

## Abstract

Antibiotics are used for disease therapeutic or preventative effects in humans and animals, as well as for enhanced feed conversion efficiency in livestock. Antibiotics can also cause undesirable effects in microbial populations, including selection for antibiotic resistance, enhanced pathogen invasion, and stimulation of horizontal gene transfer. Carbadox is a veterinary antibiotic used in the US during the starter phase of swine production for improved feed efficiency and control of swine dysentery and bacterial swine enteritis. Carbadox has been shown *in vitro* to induce phage-encoded Shiga toxin in Shiga toxin-producing *Escherichia coli* (STEC) and a phage-like element transferring antibiotic resistance genes in *Brachyspira hyodysenteriae*, but the effect of carbadox on prophages in other bacteria is unknown. This study examined carbadox exposure on prophage induction and genetic transfer in *Salmonella enterica* serovar Typhimurium, a human foodborne pathogen that frequently colonizes swine without causing disease. *S.* Typhimurium LT2 exposed to carbadox induced prophage production, resulting in bacterial cell lysis and release of virions that were visible by electron microscopy. Carbadox induction of phage-mediated gene transfer was confirmed by monitoring the transduction of a *sodCIII*::*neo* cassette in the Fels-1 prophage from LT2 to a recipient *Salmonella* strain. Furthermore, carbadox frequently induced generalized transducing phages in multidrug-resistant phage type DT104 and DT120 isolates, resulting in the transfer of chromosomal and plasmid DNA that included antibiotic resistance genes. Our research indicates that exposure of *Salmonella* to carbadox induces prophages that can transfer virulence and antibiotic resistance genes to susceptible bacterial hosts. Carbadox-induced, phage-mediated gene transfer could serve as a contributing factor in bacterial evolution during animal production, with prophages being a reservoir for bacterial fitness genes in the environment.

## Introduction

Antibiotics are used to treat bacterial infections, prevent bacterial infections, or improve feed conversion efficiency in food-producing animals. However, antibiotics have broad and unintended, sometimes called collateral, effects on microorganisms and the microbial communities they inhabit. A microorganism's response to antibiotic exposure can be monitored by gene expression signatures that indicate the organism's physiological response to antibiotic-related stress (Brazas and Hancock, 2005). Sub-minimal inhibitory concentrations of antibiotics in particular have been shown to have the unintended effect of modulating gene expression in various bacteria (Davies et al., 2006). Transcription in the foodborne pathogen *Salmonella enterica* serovar Typhimurium is affected by sub-inhibitory concentrations of antibiotics of agricultural importance, such as quinolones (Yim et al., 2011) and tetracyclines (Brunelle et al., 2013). In both studies virulence genes were among those upregulated by these antibiotics, suggesting that low concentrations of certain antibiotics may promote rather than inhibit *S*. Typhimurium survival in the host.

In the US, salmonellae are the leading cause of bacterial foodborne morbidity and mortality for humans (Scallan et al., 2011). The prevalence of multidrug-resistant *Salmonella* isolates has increased over the last few decades, and outbreak investigations indicate that antimicrobial resistant *Salmonella* isolates are associated with an increased rate of hospitalization (Varma et al., 2005a). Furthermore, patients infected with antimicrobial resistant *Salmonella* have an increased frequency of bloodstream infections and longer lengths of hospitalization (Varma et al., 2005b). Acquisition and carriage of antibiotic resistance genes by *Salmonella* is therefore a critical factor in the degree of human morbidity and mortality caused by this pathogen.

*Salmonella* has acquired antibiotic resistance genes from the environment. Its primary habitat is within the microbial community of the intestinal tract, and this community, or gut microbiota, is a reservoir for antibiotic resistance genes (Salyers et al., 2004). Sub-inhibitory antibiotics promote resistance gene transfer among gut bacteria via transposons (Shoemaker et al., 2001; Song et al., 2009), plasmids (Feld et al., 2008), and phage-like gene transfer agents (GTAs) (Stanton et al., 2008). The agricultural antibiotic carbadox is frequently used in the US during the starter phase of swine production for performance enhancement and control of enteric diseases. Carbadox is an antibacterial agent used exclusively in animals. For growth promotion and disease prophylaxis, swine feed contains 10–25 g/ton [11–28 mg/kg or parts-per-million (ppm)] and 50 g/ton [55 mg/kg (ppm)], respectively. Carbadox, a quinoxaline-di-*N*-oxide, is mutagenic, causing base pair substitutions and frameshift mutations in DNA (Beutin et al., 1981). A range of carbadox concentrations from 0.5 to 8 μg/ml (ppm) has been shown to induce prophages in Shiga toxin-producing *Escherichia coli* (STEC) (Kohler et al., 2000) and GTAs in *Brachyspira hyodysenteriae* (Stanton et al., 2008). However, it is unknown what effect carbadox would have on prophages encoded by other bacterial species, including those native to *S*. Typhimurium strains.

*Salmonella* strains have multiple prophage genomes integrated into their chromosomes. For example, the genome of *S.* Typhimurium strain LT2 contains four functional prophages: Gifsy-1 and -2 and Fels-1 and -2 (McClelland et al., 2001; Casjens, 2011). Investigation of prophages in *S.* Typhimurium indicates that many of these prophages can be induced to produce infectious virions by various environmental signals including DNA damage, antibiotics such as mitomycin C, and hydrogen peroxide (Schicklmaier et al., 1998; Figueroa-Bossi and Bossi, 1999; Schmieger and Schicklmaier, 1999; Frye et al., 2005; Garcia-Russell et al., 2009). Furthermore, prophages often encode virulence genes that enhance the pathogenesis of the bacterial strain into which the prophage is integrated (Groman, 1955; O'Brien et al., 1984; Cheetham and Katz, 1995; Waldor and Mekalanos, 1996; Figueroa-Bossi and Bossi, 1999; Mirold et al., 1999; Figueroa-Bossi et al., 2001; Ho et al., 2002; Casjens and Hendrix, 2005).

Since *Salmonella* strains usually contain multiple functional prophages and frequently colonize the swine intestinal tract, the goal of the current study was to evaluate prophage induction and genetic transfer in *S*. Typhimurium following carbadox exposure. Our research demonstrates that carbadox induced prophage production, thereby generating infectious virions capable of transferring virulence and antibiotic resistance genes via a prophage genome or generalized transduction.

## Methods

### Bacterial strains, media, and chemicals

Bacterial strains (Table 1) were grown in LB (Lennox Laboratory Supplies, Dublin, Ireland) or E minimal medium containing 0.4% glucose (Vogel and Bonner, 1956). A 5 mg/ml carbadox (Sigma-Aldrich, St. Louis, MO, USA) stock solution was made in 0.1 N NaOH and used at a final concentration of 2.5 μg/ml unless noted otherwise. Other antibiotics were used at the following concentrations: ampicillin (100 μg/ml), kanamycin (50 μg/ml), tetracycline (20 μg/ml), chloramphenicol (30 μg/ml), and carbenicillin (50 μg/ml).

**Table 1:** **Bacterial strain list**.

**Strain no**.	**Strain background**	**Genotype**	Phenotype^*^	**Source**
LT2 (BSX 1)	*S. enterica* LT2	Wild-type		John Foster
UB-0020	*S. enterica* LT2	*leuA*414, Fels2^−^, r ^−^, sup°		Miriam Susskind
UB-1731 (BSX 97)	*S. enterica* LT2 TT23657	Δ(Fels-2 Gifsy-1 Gifsy-2)		Kelly Hughes (Bunny et al., 2002)
UB-1790	*S. enterica* LT2	*leuA*414, Fels2^−^, r ^−^, sup° P22 UC-911 prophage	Kn^R^	Padilla-Meier et al., 2012
DB7004	*S. enterica* LT2	*leuA*414, supE		Winston et al., 1979
ATTC 14028s	*S. enterica*	Wild-type		Lionello Bossi (Figueroa-Bossi et al., 1997)
UK1	*S. enterica* UK1	Wild-type		John Foster
SL1344	*S. enterica* SL1344	Wild-type	Str^R^	John Foster
χ4232 (BSX 8)	*S. enterica* χ4232	Wild-type	Nal^R^	Tom Stabel
NCTC13348	*S. enterica* DT104	Wild-type	Ap^R^, Cam^R^, Tet^R^, Str^R^, Su^R^, Sp^R^	Public Health England
DT104-530	*S. enterica* DT104	Wild-type	Ap^R^, Cam^R^, Tet^R^, Str^R^	This study
DT104-745	*S. enterica* DT104	Wild-type	Ap^R^, Cam^R^, Tet^R^, Str^R^, Kn^R^	This study
DT104b-5414	*S. enterica* DT104	Wild-type	Ap^R^, Cam^R^, Tet^R^, Str^R^	This study
DT120-150	*S. enterica* DT120	Wild-type	Ap^R^, Cam^R^, Tet^R^, Str^R^	This study
DT120-305	*S. enterica* DT120	Wild-type	Ap^R^, Cam^R^, Tet^R^, Str^R^	This study
DT120-613	*S. enterica* DT120	Wild-type	Ap^R^, Cam^R^, Tet^R^, Str^R^	This study
DT120-7055	*S. enterica* DT120	Wild-type		This study
DT193-1434	*S. enterica* DT193	Wild-type	Ap^R^, Cam^R^, Tet^R^, Str^R^, Kn^R^, Nal^R^	This study
DT208-2348	*S. enterica* DT208	Wild-type	Ap^R^, Cam^R^, Tet^R^, Nal^R^	This study
U302-4715	*S. enterica* U302	Wild-type	Ap^R^, Cam^R^, Tet^R^,	This study
BSX 7	*S. enterica* TT22971 (LT2)	*metA22 metE551 trpD2 ilv-452 leu pto* (leaky) *hsdLT6 hsdSA29 hsdB strA120*/pKD46	Ap^R^, 30°C	John Roth via Max Wu
BBS 119	*S. enterica* LT2	*metA22 metE551 trpD2 ilv-452 leu pto* (leaky) *hsdLT6 hsdSA29 hsdB strA120*		BSX 7 cured of pKD46
BBS 120	*S. enterica* LT2	*metA22 metE551 trpD2 ilv-452 leu pto* (leaky) *hsdLT6 hsdSA29 hsdB strA120*/pCP20	Ap^R^, 30°C	BBS 119/pCP20
BBS 231	*S. enterica* LT2	*metA22 metE551 trpD2 ilv-452 leu pto (leaky) hsdLT6 hsdSA29 hsdB strA120 hisDCBHA::neo*	Kn^R^	BSX 7/oBBI 197/198 (*hisD-A::neo*)
BBS 233	*S. enterica* χ4232	*hisDCBHA::neo*	Nal^R^, Kn^R^	BSX 8 × HT BBS231
BBS 243	*S. enterica* χ4232	Δ*hisDCBHA*	Nal^R^	BBS 233 × HT BBS 120
BBS 561	*S. enterica* LT2	*metA22 metE551 trpD2 ilv-452 leu pto* (leaky) *hsdLT6 hsdSA29 hsdB strA120 sodCIII*::*neo*	Kn^R^	BSX 7/oBBI 300/301 neo
BBS 565	*S. enterica* LT2	*sodCIII*::*neo*	Kn^R^	BSX 1 × HT BBS 561
BBS 649	*S. enterica* DT104-745	Δ*floR* Δ*tet* Δ*pse-1*	Kn^R^	
BBS 651	*S. enterica* DT104-745	Δ*floR* Δ*tet* Δ*pse-1*/pKD46	Kn^R^, Ap^R^, 30°C	BBS 649/pKD46
BBS 998	*S. enterica* UB-1731	*sodCIII*::*neo*	Kn^R^	BSX 97 × HT BBS 561
BBS 1004	*S. enterica* LT2	*metA22 metE551 trpD2 ilv-452 leu pto* (leaky) *hsdLT6 hsdSA29 hsdB strA120* Fels-1::*neo*	Kn^R^	BSX 7/oBBI 302/439 *neo*
BBS 1008	*S. enterica* LT2	Δ(Fels-2 Gifsy-1 Gifsy-2) Fels-1::*neo*	Kn^R^	BSX 97 × HT BBS 1004
BBS 1010	*S. enterica* DT104-745	Δ*floR* Δ*tet* Δ*pse-1 hisDCBHA::cat*	Kn^R^, Cam^R^	BBS 651/oBBI 197/198 *cat*
BBS 1012	*S. enterica* DT104-745	Δ*floR* Δ*tet* Δ*pse-1* Δ*hisDCBHA*	Kn^R^	BBS 1010/pCP20
UB-1703	*E. coli* K-12	λ prophage		Roger Hendrix (Hendrix and Duda, 1992)
UB-1704	*E. coli* K-12	HK97 prophage		Roger Hendrix
594	*E. coli* K-12 594		Str^R^	Weigle, 1966
UB-1458	*S. flexneri* PE577	Wild-type		Renato Morona (Casjens et al., 2004)
UB-1496	*S. flexneri* PE577	Sf6 prophage		Renato Morona

* Known antibiotic resistance phenotypes. Ap, Ampicillin; Cam, Chloramphenicol; Tet Tetracycline; Str, Streptomycin; Su, Sulfamethoxazole; Sp, Spectinomycin; Kn, Kanamycin; Nal, Nalidixic acid.

### *S*. Typhimurium gene and prophage knockouts by recombineering

Oligonucleotide primers for PCR amplification and construction of gene and prophage knockouts are listed in Table 2. *S.* Typhimurium gene and prophage knockouts were constructed by recombineering (recombination-mediated genetic engineering) as previously described (Bearson and Bearson, 2008; Bearson et al., 2008). Briefly, the 5' end of a gene knockout primer (bold, Table 2) has homology to 32–44 bp of the target gene whereas the 3' end contains universal sequences (underlined) to amplify an antibiotic resistance gene and truncate potential translation of the target gene. A gene knockout primer set was used to PCR amplify either the *neo* or the *cat* gene. Gel electrophoresis was performed on the amplification product of a knockout fragment and the respective DNA fragment was gel extracted using a Freeze'n Squeeze column (Bio-Rad, Hercules, CA). Each knockout fragment was transformed (Sambrook and Russell, 2006) into an arabinose-induced *S.* Typhimurium strain containing the pKD46 plasmid (Datsenko and Wanner, 2000). Transformants containing the knockout were selected on LB agar medium containing kanamycin. If necessary, the gene knockout with the *neo* marker was moved to another strain background by transduction using a P22 phage with a high transduction frequency. Flp mediated deletion of the *neo* or *cat* gene was performed by transferring the pCP20 plasmid into the knockout strain by either transduction or transformation followed by a procedure to screen for loss of resistance to kanamycin (Cherepanov and Wackernagel, 1995).

**Table 2:** **Primers used in this study**.

**Gene/phage target**	**Primer**	**Sequence (5'–3')**	**References**
*hisD-A*	oBBI 197	**ctgatggcgctgcgcttatcaggcctacgtaatgc**atagagcagtgacgtagtcgc	This study
	oBBI 198	**cgttttgccagcattggatggcctccttaacg**atagctgaatgagtgacgtgc	
*sodCIII*	oBBI 300	**gttaaccttgtaaatgccaatggcacaggtcaaaagatcg**atagctgaatgagtgacgtgc	This study
	oBBI 301	**gtggaacagtgcctcacagagtgaattttattttataacgc**atagagcagtgacgtagtcgc	
Fels-1	oBBI 302	**cattcattaaggaaggaaagagtatgactgtagaaaaatccg**atagctgaatgagtgacgtgc	This study
	oBBI 439	**cataaccacttaacatcttgttttatctaaataaaattaagcat**agagcagtgacgtagtcgc	
P22-like	ST104Gp1F	gacgcccgtcactgcacagtta	This study
	ST104Gp1R	acccggcgacgcttaatctg	

Bold text indicates homology to the target gene/phage.

Underlined text indicates sequence for neo or cat amplification.

### Determination of phage titers following carbadox induction of bacterial strains lysogenized with the model phages P22, λ, HK97, and SF6

Bacterial strains lysogenized with P22 (UB-1790), λ (UB-1703), HK97 (UB-1704), and Sf6 (UB-1496) were grown in LB broth at 37°C with shaking. At a density of 1 × 10^8^ bacterial cells/ml, carbadox was added to a final concentration of 0.5 μg/ml (ppm). At the indicated times after carbadox addition, aliquots of the cultures were removed, shaken with several drops of chloroform to complete lysis, and titered on the permissive host. Strains used to titer the phage lysates were DB7004 (P22), 594 (λ and HK97), and UB-1458 (Sf6).

### Carbadox induction of wild-type *S*. Typhimurium

*S.* Typhimurium strains were grown in LB 0.4% glucose at 37°C with shaking at 180 rpm. Carbadox was added to cultures at OD_600_ = 0.2 at a final concentration of 2.5 μg/ml (ppm). Incubation of cultures was continued to monitor for bacterial cellular lysis.

### Phage transduction using carbadox-induced *S*. Typhimurium lysates

Supernatants from non-induced and carbadox-induced bacterial cultures were harvested by adding 100 μl of chloroform, vortexing gently, and allowing the culture to incubate for ~15 min with shaking. The cultures were centrifuged at 1000× g for 20 min and the supernatant was transferred to a fresh tube containing 200 μl of chloroform for storage. Bacterial lysates were plated to LB to ensure that viable bacterial cells were not present. An overnight culture of the transduction recipient was grown in LB or LB glucose at 37°C with shaking. The transduction was performed with equal volumes of both the *S.* Typhimurium recipient strain and either the non-induced or the carbadox-induced culture supernatant. The transduction was incubated at 37°C for ~1–3 h before plating on the appropriate selective medium. To determine the transduction frequency for transfer of the histidine operon, the transduction was plated onto E glucose minimal medium. Transduction frequency for antibiotic resistance gene transfer (*sodCIII*:: neo in Fels-1, plasmid-encoded kanamycin, and chromosomally encoded tetracycline) was determined by plating the transduction to LB containing either kanamycin or tetracycline.

### Phage purification for electron microscopy

Overnight cultures were diluted 1:100 in 400 ml E glucose minimal medium and incubated at 37°C with shaking. At OD_600_ = 0.5, carbadox was added to a final concentration of 2.5 μg/ml, and incubation continued until lysis was achieved. Phages were purified and visualized by electron microscopy as described previously (Humphrey et al., 1997) with the following modifications. Purified phages were negatively stained by mixing phage samples with an equal volume of phosphotungstic acid (2%, pH 7.0). Samples were deposited on Formvar-coated 200-mesh carbon-reinforced copper grids (Electron Microscopy Sciences, Hatfield, PA) and viewed with a FEI Tecnai G2 BioTWIN electron microscope (80 kV; Hillsboro, OR).

## Results and discussion

### Carbadox induces *S*. Typhimurium and other *enterobacteriaceae* prophages to cause phage replication and cell lysis

**Figure 1:**
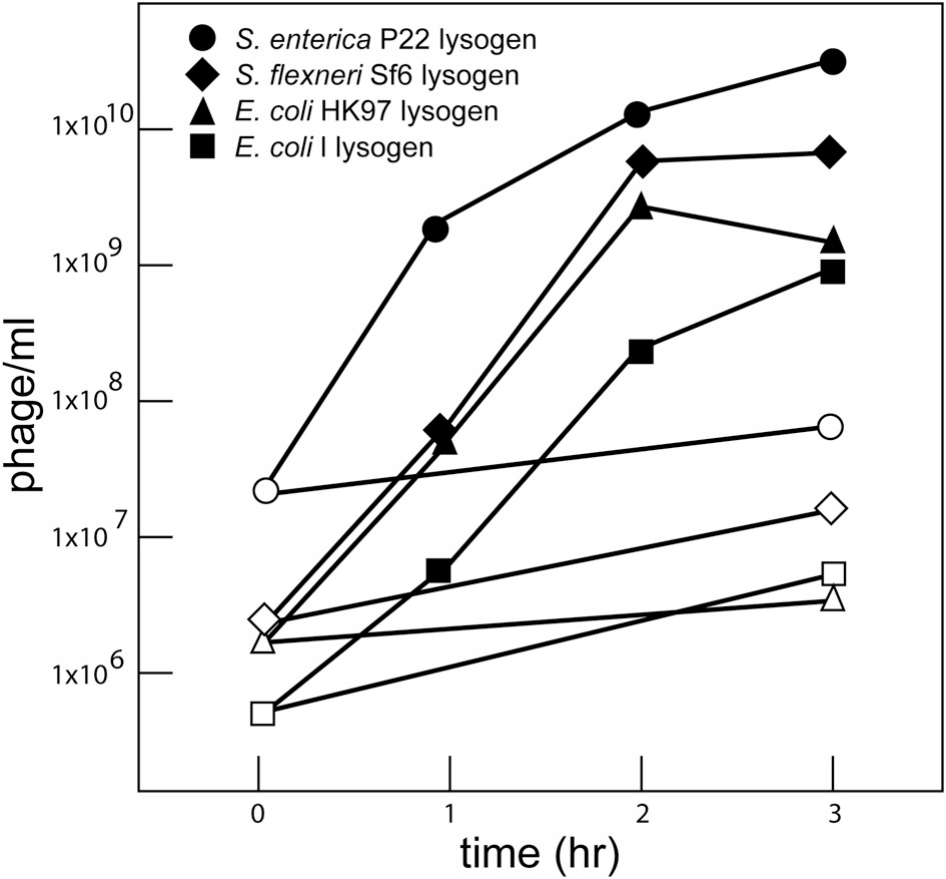
**Carbadox induction of *Enterobacteriaeae* prophages**. Bacterial strains lysogenized with P22 (UB-1790), λ (UB-1703), HK97 (UB-1704), and Sf6 (UB-1496) were grown in LB broth at 37°C with shaking. At a density of 1 × 10^8^ bacterial cells/ml, carbadox was added to a final concentration of 0.5 μg/ml. Phage lysates were obtained by shaking with chloroform at the indicated times after carbadox addition and titered on a permissive host. Open symbols indicate cultures with no added carbadox and closed symbols indicate cultures with carbadox added. The different induced lysogens are indicated in the figure insert.

To test whether well-characterized prophages in *S. enterica* and other *Enterobacteriaceae* species are induced by carbadox, we monitored the infectious phages produced by carbadox-treated cultures of bacterial strains that were lysogenized by the “model system” phages P22 (*S. enterica*), λ and HK97 (*E. coli*), and Sf6 (*Shigella flexneri*). In each case, at least partial clearing of the culture indicated a substantial fraction of the cells in the culture had lysed by about 2 h. All four prophages gave an approximately three-log increase in free phage in the culture (Figure 1), with about 10–200 progeny phage produced per bacterium that were initially present at the time of carbadox addition. Several concentrations of carbadox were tested, and 0.5 μg/ml (ppm) is shown as the minimum that gave good induction of P22. Carbadox induces the P22 *Salmonella* prophage as well as *E. coli* and *S. flexneri* prophages under these conditions, and this is not surprising given its apparent mechanistic similarities to the action of mitomycin C. Carbadox is a DNA damaging agent, and DNA damage induced by mitomycin C is known to induce the bacterial SOS pathway, which induces prophages. It is likely capable of induction of prophages from many if not all *Enterobacteriaceae* bacterial species, as well as more distantly related bacterial phyla.

**Figure 2:**
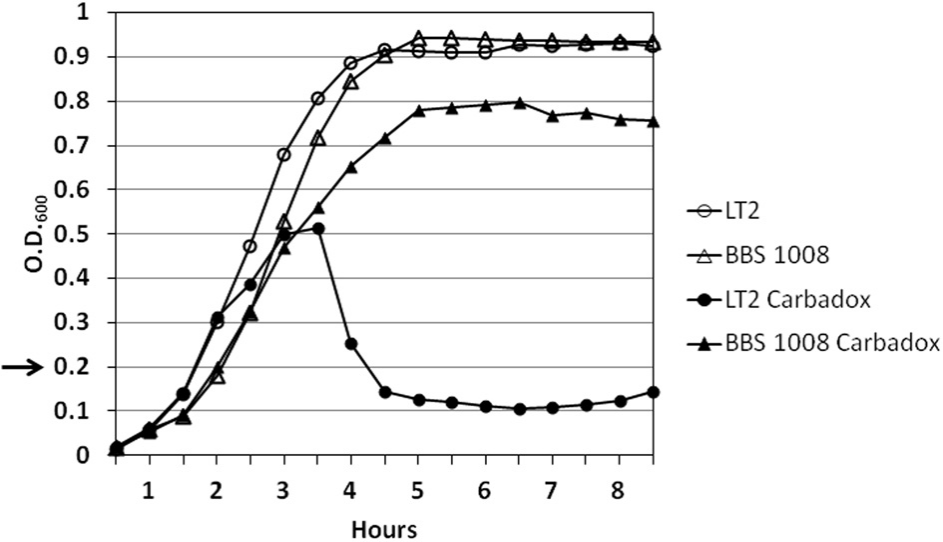
**Carbadox exposure of wild-type *S.* Typhimurium LT2 results in bacterial cell lysis**. *S.* Typhimurium strains (LT2 and BBS 1008) were grown in LB glucose medium at 37°C with shaking. At OD_600_ = 0.2 (arrow), carbadox (2.5 μg/ml) was added to cultures indicated by the closed symbols. The open symbols indicate control cultures without carbadox.

To examine prophage induction by carbadox in wild-type *S.* Typhimurium isolates containing their natural prophages, we initially examined *S.* Typhimurium LT2, a strain that is widely used in the study of *Salmonella* genetics in the laboratory (Lilleengen, 1948). Cultures of wild-type LT2 in early log phase growth were exposed to carbadox. The bacterial density of the culture abruptly decreased at ~2 h following exposure to 2.5 μg/ml (ppm) of carbadox (Figure 2). Mitomycin C exposure is known to result in the induction of prophage Fels-1 from strain LT2 (Garcia-Russell et al., 2009). The fact that carbadox induces wild-type phage λ (above), which is only known to be induced by the SOS function of activated RecA protein (Little, 1993), strongly supports this mechanism for carbadox-mediated induction. Thus, the decrease in LT2 cell density is almost certainly due to bacterial cell lysis resulting from prophage induction.

LT2 is known to carry four prophages. Amplification of the integrase gene from DNA extracted from phage heads indicated that at least one of these, the Fels-1 prophage, was induced following carbadox exposure (Stanton and Humphrey, unpublished data). The BBS 1008 strain is an LT2 derivative from which all four prophages have been deleted. Following exposure of BBS 1008 to 2.5 μg/ml of carbadox, the bacterial culture density did not decrease (Figure 2), indicating that these prophages are responsible for the bacterial cell lysis phenotype induced by carbadox exposure.

Examination of the purified phages by electron microscopy demonstrated the presence of mostly empty phage heads in the carbadox-induced culture of LT2 (data not shown). Treatment of *S.* Typhimurium LT2 and other strains with mitomycin C is known to induce Fels-1 plaque-forming phages with poor efficiency (Figueroa-Bossi and Bossi, 1999), so it is perhaps not surprising that whole phage particles were not seen. Nonetheless, phage heads were either greatly reduced or were not observed in the absence of carbadox for the LT2 strain and in the presence of carbadox for BBS 1008. These results confirm prophage induction in a natural wild-type *S.* Typhimurium isolate following carbadox exposure.

### Carbadox exposure of *S.* Typhimurium LT2 promotes phage transduction into a susceptible bacterial host

To monitor phage transduction frequency, a *neo* gene was inserted by recombineering into the putative virulence factor *sodCIII* on the Fels-1 prophage of strain LT2 to create strain *S.* Typhimurium BBS 565. The carbadox-induced phage lysate from BBS 565 (LT2 *sodCIII*::*neo*) efficiently transduced the kanamycin-sensitive strain BBS 243, as demonstrated by 8 × 10^3^ kanamycin-resistant transductants per ml of lysate. In the absence of carbadox induction, BBS 243 remained susceptible to kanamycin following transduction with the control supernatant from BBS 565. This indicates that carbadox-induced prophages can carry genetic material from a donor into a recipient bacterial strain.

### Carbadox-induction of multidrug-resistant *S*. Typhimurium DT104 results in generalized transduction of chromosomal and plasmid DNA

Generalized transduction involves the packaging of random host DNA (genomic or plasmid) into a bacteriophage particle and the transfer of that DNA to a recipient strain. Bacteriophage P22 is a generalized transducing phage that is commonly used for genetic experiments with *Salmonella* (Zinder and Lederberg, 1952; Kropinski et al., 2007). Although *S.* Typhimurium LT2 contains multiple prophages, it does not contain a P22-like prophage that performs generalized transduction. Some multidrug-resistant *S.* Typhimurium isolates like phage type DT104 harbor a P22-like prophage. This prophage has been described as PDT17, ST104, and prophage 1 (Schmieger and Schicklmaier, 1999; Tanaka et al., 2004; Cooke et al., 2008). To determine whether carbadox exposure could induce generalized transduction, *S.* Typhimurium DT104 NCTC13348 was exposed to carbadox. The culture lysed, and the phage-containing supernatant was harvested. The BBS 243 strain, a histidine auxotroph lacking the genes *hisDCBHA*, could be successfully transduced to *his*^+^ with this lysate. Generalized transduction was observed by growth on minimal medium lacking histidine, demonstrating the transfer of the *his* operon from the *his*^+^ donor (DT104) to the *his*^−^ recipient (BBS 243) (Table 3). The results show that carbadox exposure of multidrug-resistant *S.* Typhimurium DT104 can stimulate generalized transduction and therefore chromosomal gene transfer.

**Table 3:** **Average frequency of generalized transduction per 0.5 ml of lysate from numerous *S.* Typhimurium donor strains into BBS 243**.

***Salmonella* donor strain**	**Not induced**	**Carbadox induced ± s.e.m**.
LT2	0	0
UB-1731	0	0
ATTC 14028s	0	0
UK1	0	0
SL1344	0	0
χ4232	0	0
DT104 (NCTC13348)	0	6 ± 1.7
DT104-530	0	291 ± 100.8
DT104b-5414	<1	117 ± 52.8
DT120-150	0	124 ± 19.3
DT120-305	<1	420 ± 94.8
DT120-613	<1	86 ± 16.7
DT120-7055	0	0
DT193-1434	0	0
DT208-2348	0	0
U302-4715	0	0

The *Salmonella* genomic island-1 (SGI-1) is ~43 kb and typically contains chromosomally-encoded resistance genes for multiple antibiotics including ampicillin, chloramphenicol, and tetracycline within an integron (Boyd et al., 2001; Mulvey et al., 2006). We attempted to transduce SGI-1 but were unsuccessful. Due to the size of SGI-1, transduction of this entire island by P22 into another *Salmonella* strain that does not already contain a segment of SGI-1 is inefficient [P22 packages about 43.4 Kbp of DNA (Kropinski et al., 2007)]. To overcome this experimental limitation, we utilized a DT104 derivative (BBS 1012) as our recipient strain. The BBS 1012 strain is a histidine auxotroph that has an internal deletion within SGI-1, resulting in sensitivity to ampicillin, chloramphenicol, and tetracycline due to the loss of multiple antibiotic resistance genes. In addition, BBS 1012 contains a natural plasmid that confers kanamycin resistance. Transduction of BBS 1012 with the carbadox-induced phage lysate from wild-type *S.* Typhimurium DT104-530 (kanamycin sensitive) resulted in the transfer of tetracycline resistance following selection on tetracycline-containing LB agar medium. Transduction into the BBS 1012 strain was confirmed by growth on medium containing kanamycin and the absence of growth on minimal medium without histidine. Furthermore, the initial selection of BBS 1012 on LB medium containing tetracycline resulted in 100% co-transduction of resistance to both chloramphenicol and carbenicillin. The *floR* and *pse-1* genes encode resistance to chloramphenicol and carbenicillin, respectively, and these genes are located adjacent to *tetG* on SGI-1. These results indicate that exposing multidrug-resistant *S.* Typhimurium DT104 to carbadox can promote the transfer of numerous genes co-located within SGI-1 that encode resistance to multiple classes of antibiotics. Thus generalized transduction could participate in bacterial strain evolution by providing an assortment of antibiotic resistance genes for recombination within this important genomic region.

Although the SGI-1 integron in *S.* Typhimurium DT104 encodes multiple antibiotic resistance genes, some strains contain additional antibiotic resistance genes encoded on plasmids. Transduction of BBS 243 (kanamycin sensitive) with the carbadox-induced phage lysate from *S.* Typhimurium DT104 (745) resulted in bacterial growth on LB medium containing kanamycin, demonstrating the transfer of the plasmid encoding kanamycin resistance. Thus, carbadox exposure promoted generalized transduction of this natural plasmid as well as chromosomally-encoded antibiotic resistance genes in multidrug-resistant *S.* Typhimurium DT104.

### Carbadox-induced gene transfer is a general phenomenon that occurs in multidrug-resistant *S*. Typhimurium strains DT120 and DT104

Since the prevalence of multidrug-resistant *S.* Typhimurium strains has increased over the last few decades, we wanted to determine whether carbadox-induced gene transfer is unique to *S.* Typhimurium DT104 or is a general property of multidrug-resistant *S.* Typhimurium strains. Phage lysates were harvested from several *S.* Typhimurium phage types following carbadox exposure and used to transduce the recipient strain BBS 243 (Δ*hisDCBHA*) with selection on E glucose minimal medium; several different phage types were investigated as these, by definition (i.e., DT), should have varying prophage content. Carbadox-induced phage lysates from several *S.* Typhimurium DT104 and DT120 isolates resulted in growth on minimal medium, indicating the transfer of the *his* operon to BBS 243 (Table 3). The results suggest that generalized transduction following carbadox induction is a common phenomenon for multidrug-resistant *S.* Typhimurium DT104 and DT120.

We PCR amplified the P22 gene *1* (encoding gp1/portal protein) from several of the DT104 and DT120 isolates that we have shown are capable of generalized transduction, suggesting that these multidrug-resistant isolates contain a P22-like prophage. To confirm that a phage capable of generalized transduction is required for carbadox-induced gene transfer, the P22-like prophage (prophage 1) was deleted from DT104 using recombineering. Gene transfer into BBS 243 was eliminated following transduction with a carbadox-induced phage lysate from the DT104 P22-like prophage knockout strain, indicating that the P22-like prophage is responsible for the generalized transduction from *S.* Typhimurium DT104. In support of this, the *S.* Typhimurium strains LT2, UK1, SL1344, and χ4232 (frequently investigated strains in the literature) are incapable of generalized transduction and do not contain a P22-like prophage. In contrast, genome scanning has identified integrated P22-like prophages in the genome sequences of isolates of *S. enterica* serovars Arizonae, Cholerae, Dublin, Hadar, Heidelberg, Houtenae, Johannesburg, Mississippi, Montevideo, Newport, Paratyphi, Rubislaw, Schwarzengrund, Tennessee, Typhimurium, Uganda, Wandsworth, and Welteverden; this suggests that P22-like prophages are common among *Salmonella* serovars and the potential for strain evolution due to generalized transduction is perhaps underappreciated. The capability of generalized transduction among *Salmonella* serovars is reinforced with the knowledge that P22 phage lysates are known to be stable for many years in the laboratory. Likewise, an ecological significance of phages in the environment is that DNA encapsulated within a phage head is protected from nucleases and therefore can survive outside of a bacterial host until encountering a recipient.

Swine environments, including swine manure, have been shown to contain abundant phage populations (McLaughlin et al., 2006; Wang et al., 2010). Bacteriophage populations present in manure could be derived principally from prophage induction of bacteria present in manure or a combination of induction from within the swine gastrointestinal tract and in manure. Prophage induction can be stimulated by various environmental signals and stresses including ultraviolet light, hydrogen peroxide, mitomycin C, and carbadox. Analysis of fecal phage metagenomes from medicated swine administered in-feed antibiotics [carbadox or ASP250 (chlortetracycline, sulfamethazine, and penicillin)] compared to non-medicated swine suggested that prophages were induced with antibiotic treatment (Allen et al., 2011). Similar work with mouse fecal phage metagenomes has shown that antibiotic treatment caused an increase in the abundance of phage-encoded antibiotic resistance genes (Modi et al., 2013). This suggests that antibiotic-induced phage-mediated transduction may contribute to antibiotic resistance gene transfer during animal production. Relatively little information is available concerning the extent of carbadox-induced prophage from bacteria, as *Salmonella* is only the third bacterial genus for which this response has been described (Kohler et al., 2000; Stanton et al., 2008). Additional information is needed to understand the capacity for carbadox to induce prophages during swine production since there is a potential for dissemination into the environment following manure application onto agricultural soils.

## Conclusions

Prophages are a potential environmental reservoir for bacterial fitness genes and may drive the emergence of new epidemic clones (Brussow et al., 2004). Prophages integrated in the genomes of *Salmonella* strains can encode genes associated with virulence or antimicrobial resistance (Figueroa-Bossi and Bossi, 1999; Figueroa-Bossi et al., 2001; Moreno Switt et al., 2013). Therefore, the pathogenic potential of a particular *Salmonella* strain depends in part upon the prophage repertoire integrated into the bacterial genome, and acquisition of prophages could conceivably result in enhanced bacterial virulence or survival during host colonization. In this report, we demonstrate that exposure of several different *S.* Typhimurium isolates to the agricultural antibiotic carbadox resulted in the production of transducing particles capable of transferring the individual phage genome as well as chromosomal and plasmid DNA by generalized transduction.

## Author contributions

Conceived and designed experiments: Bradley L. Bearson, Heather K. Allen, Sherwood R. Casjens, Thaddeus B. Stanton, and Brian W. Brunelle. Performed the experiments: Bradley L. Bearson, Heather K. Allen, Sherwood R. Casjens, In S. Lee, Brian W. Brunelle, and Thaddeus B. Stanton. Wrote and edited the manuscript: Bradley L. Bearson, Heather K. Allen, Sherwood R. Casjens, Brian W. Brunelle, and Thaddeus B. Stanton.

### Conflict of interest statement

The authors declare that the research was conducted in the absence of any commercial or financial relationships that could be construed as a potential conflict of interest.
